# Supplementary material for: SPARClink: an interactive tool to visualize the impact of the SPARC program
Source: F1000Res. 2022 Jan 31;11:124. [Version 1] doi: 10.12688/f1000research.75071.1 (PMC9936100; doi:10.12688/f1000research.75071.1)
Supplement: Peer review history

# Peer review: Reviewer response for version 1

**DOI:** 10.5256/f1000research.78888.r160993

**Published:** 2023-02-16

**Authors:** Angela Pinot de Moira

**Affiliations:** 1 Section of Epidemiology, University of Copenhagen, Copenhagen, Denmark; 2 National Heart and Lung Institute, Imperial College London, London, UK

This is a clearly written paper describing SPARClink, a web tool that queries all external publications and creates a database and visualisations of publications that have utilised SPARC-funded resources, including datasets, publications and protocols. As well as documenting the impact of the SPARC consortium, the tool will be an invaluable resource for researchers utilising SPARC outputs for their research.

 My main comment to the paper is regarding the aim to demonstrate the benefits of the FAIR principles. Currently, the paper mainly focuses on the retrieval of any publication utilising SPARC resources, i.e. including publications directly funded by SPARC. To demonstrate how the tool can be used to highlight/quantify the benefits of the FAIR principles, it would be useful to provide examples of this in the paper, i.e. demonstrate the reuse of SPARC data. If I have understood correctly, this is possible by filtering extracted publications on whether they are SPARC-funded or non-SPARC funded. It would be useful to provide a visualization with the non-SPARC funded filter applied, so that the extent of data reuse can be seen.

 My second suggestion is to include a box of key terminology in the paper. There are a lot of terms that may not be understood by the reader and although links to definitions are provided, a glossary box may aid reading.

Are the conclusions about the tool and its performance adequately supported by the findings presented in the article?

Partly

Is the rationale for developing the new software tool clearly explained?

Yes

Is the description of the software tool technically sound?

Yes

Are sufficient details of the code, methods and analysis (if applicable) provided to allow replication of the software development and its use by others?

Yes

Is sufficient information provided to allow interpretation of the expected output datasets and any results generated using the tool?

Partly

Reviewer Expertise:

Epidemiology, FAIR principles

I confirm that I have read this submission and believe that I have an appropriate level of expertise to confirm that it is of an acceptable scientific standard.

# Peer review: Reviewer response for version 1

**DOI:** 10.5256/f1000research.78888.r160964

**Published:** 2023-01-26

**Authors:** Karim Fouad, Abel Torres-Espin

**Affiliations:** 1 University of Alberta, Edmonton, Canada; 2 University of California San Francisco, San Francisco, CA, USA

This is a very succinct article introducing a web tool for querying and visualizing the scientific output of the SPARC program and how it has been utilized by the research community. The process begins with extracting and storing related data via APIs. Then word clouds and key word searches allow users to refine their search to then visualize the relation between the SPARC output products. The product is a very useful tool to explore the ongoing impact and use of SPARC related research and products. Potentially the impact of the tool is not really on what the authors mention (i.e., show the impact of FAIR data sharing on the overall scientific community) and thus somewhat overstated. The visualization tool simply provides a summary of the reuse of SPARC related work products, but not a comparison to other approaches for data sharing that would allow for assessing the impact of FAIR. The strength lies in the demonstration of data reuse, specific impact of their data sets, etc. This is a highly valuable tool on so many levels including strategies for future research and general education of the value of data sharing.

 The manuscript would benefit from a few clarifications and details especially in the figure legends. For example, the difference between a SPARC publication and a data set should be explained. Are SPARC data sets not published?

 Figure 3 show a lot of white space, and the legend would benefit from more detail. What does define the size of the nodes in the visualization? Did the authors consider directional links between the items in the form of a directed graph, which would simplify comprehension of what is shown? For example, the edge of the graph could show the direction from the node citing to the one receptor of a citation.

 Figure 4 requires a legend for the different colors, an explanation for the different size of the nodes, and once again would benefit from directional edges. Lastly, not surprisingly the overwhelming links to publications mask the reuse of data sets. Maybe a different filter could be applied to show that important relation.

 Minor suggestions:

- In the Abstract, consider adding ‘electrical’ in front of neuromodulation.
- 3d paragraph in introduction, consider not to refer (i.e., these tools) when starting a new paragraph.
- Please define “citations of SPARC-funded resources”.

Are the conclusions about the tool and its performance adequately supported by the findings presented in the article?

Partly

Is the rationale for developing the new software tool clearly explained?

Yes

Is the description of the software tool technically sound?

Yes

Are sufficient details of the code, methods and analysis (if applicable) provided to allow replication of the software development and its use by others?

Yes

Is sufficient information provided to allow interpretation of the expected output datasets and any results generated using the tool?

Partly

Reviewer Expertise:

Neuroplasticity, data sharing

We confirm that we have read this submission and believe that we have an appropriate level of expertise to confirm that it is of an acceptable scientific standard.

# Peer review: Reviewer response for version 1

**DOI:** 10.5256/f1000research.78888.r140292

**Published:** 2022-06-27

**Authors:** Tao Zeng

**Affiliations:** 1 AS Key Laboratory of Computational Biology, Bio-Med Big Data Center, University of Chinese Academy of Sciences, Shanghai, China

In the paper “SPARClink: an interactive tool to visualize the impact of the SPARC program”, authors aim to introduce a web tool SPARClink for visualizing the impact of SPARC, whose methods and outcomes support FAIR guidelines.

 SPARClink should be a useful tool/software for supporting SPARC program and corresponding consortium. For the work and introduction in this paper, I have several suggestions:

1. SPARC program employs FAIR, so, it is necessary to introduce more about corresponding function of SPARClink serving each FAIR guideline, e.g. for “Reusable”, what data or protocol can be reused and how other researchers can obtain and reuse them.
2. Similar to other programs, the data produced in SPARC would have raw data, pre-processed data, or analyzed data, or summary data, etc. The organization or level of SPARC data is better to be clearly introduced, and supply detailed cases of how SPARClink can manage and share these data.
3. In current implementation, “interactive force-based undirected graph visualization” is simple, and the directed relation is better to consider, e.g. the paper and its public data, and the paper and its reused data, would have different relation directions. Also, in the abstract, the authors stated “a novel knowledge graph-based structure was created to visualize …” Thus, the novelty of the network structure and representation should have highlights in revision.
4. For interactive visualization shown in Figure 4, or used in current web tool, the network has less information - it is better to directly show the information about each node or edge on network in web page in a scalable manner, especially for large knowledge network.
5. The word maps shown in Figure 5 would be a general function in many other tools, it is necessary to offer the interesting word or sentence association between key words and SPARC outcomes.
6. Indeed, for FAIR, the software SPARClink itself is suggested to supply Docker version, so other users can easily reuse such useful framework into their applications.

Are the conclusions about the tool and its performance adequately supported by the findings presented in the article?

Partly

Is the rationale for developing the new software tool clearly explained?

Yes

Is the description of the software tool technically sound?

Yes

Are sufficient details of the code, methods and analysis (if applicable) provided to allow replication of the software development and its use by others?

Partly

Is sufficient information provided to allow interpretation of the expected output datasets and any results generated using the tool?

Yes

Reviewer Expertise:

Machine learning and bioinformatics

I confirm that I have read this submission and believe that I have an appropriate level of expertise to confirm that it is of an acceptable scientific standard, however I have significant reservations, as outlined above.
